# Precise Genome Editing in miRNA Target Site via Gene Targeting and Subsequent Single-Strand-Annealing-Mediated Excision of the Marker Gene in Plants

**DOI:** 10.3389/fgeed.2020.617713

**Published:** 2021-01-12

**Authors:** Namie Ohtsuki, Keiko Kizawa, Akiko Mori, Ayako Nishizawa-Yokoi, Takao Komatsuda, Hitoshi Yoshida, Katsuyuki Hayakawa, Seiichi Toki, Hiroaki Saika

**Affiliations:** ^1^Institute of Agrobiological Sciences, National Agriculture and Food Research Organization (NARO), Tsukuba, Japan; ^2^Nisshin Flour Milling Inc., Tsukuba, Japan; ^3^Japan Science and Technology Agency (JST), Precursory Research for Embryonic Science and Technology (PRESTO), Kawaguchi, Japan; ^4^Institute of Crop Sciences, NARO, Tsukuba, Japan; ^5^Graduate School of Nanobioscience, Yokohama City University, Yokohama, Japan; ^6^Kihara Institute for Biological Research, Yokohama City University, Yokohama, Japan

**Keywords:** gene targeting, precise genome modification, single-strand annealing, cleistogamy 1, *oryza sativa*, miRNA target site

## Abstract

Gene targeting (GT) enables precise genome modification—e.g., the introduction of base substitutions—using donor DNA as a template. Combined with clean excision of the selection marker used to select GT cells, GT is expected to become a standard, generally applicable, base editing system. Previously, we demonstrated marker excision via a *piggyBac* transposon from GT-modified loci in rice. However, *piggyBac*-mediated marker excision has the limitation that it recognizes only the sequence TTAA. Recently, we proposed a novel and universal precise genome editing system consisting of GT with subsequent single-strand annealing (SSA)-mediated marker excision, which has, in principle, no limitation of target sequences. In this study, we introduced base substitutions into the microRNA miR172 target site of the *OsCly1* gene—an ortholog of the barley *Cleistogamy1* gene involved in cleistogamous flowering. To ensure efficient SSA, the GT vector harbors 1.2-kb overlapped sequences at both ends of a selection marker. The frequency of positive–negative selection-mediated GT using the vector with overlapped sequences was comparable with that achieved using vectors for *piggyBac*-mediated marker excision without overlapped sequences, with the frequency of SSA-mediated marker excision calculated as ~40% in the T_0_ generation. This frequency is thought to be adequate to produce marker-free cells, although it is lower than that achieved with *piggyBac*-mediated marker excision, which approaches 100%. To date, introduction of precise substitutions in discontinuous multiple bases of a targeted gene using base editors and the prime editing system based on CRISPR/Cas9 has been quite difficult. Here, using GT and our SSA-mediated marker excision system, we succeeded in the precise base substitution not only of single bases but also of artificial discontinuous multiple bases in the miR172 target site of the *OsCly1* gene. Precise base substitution of miRNA target sites in target genes using this precise genome editing system will be a powerful tool in the production of valuable crops with improved traits.

## Introduction

Biological species have developed repair systems for DNA double-strand breaks (DSBs) as such repairs are critical to life. DSB repair systems have been classified traditionally into two pathways: non-homologous end joining (NHEJ) and homologous recombination (HR) (Chapman et al., 2012; Hustedt and Durocher, 2016). The former is a rapid but error-prone response that results in some inserted and/or deleted bases due to the simple ligation of both ends of a DSB site. The latter is an accurate repair system that uses a homologous region of the sister chromatid as a template at the DSB site.

Gene targeting (GT) is a powerful genome engineering technology that can be used to introduce various types of mutation into a target gene locus by HR using a donor DNA as a template. The first demonstration of GT in higher plants was reported as far back as 1988 (Paszkowski et al., 1988). Much later, a GT procedure applied to an endogenous gene was first reported in the *WAXY* gene in rice (Terada et al., 2002). Since then, knock-out as well as knock-in mutants of several genes have been produced using GT techniques (Shimatani et al., 2015). Although the CRISPR/Cas9 system is now used commonly for gene knock-out in various plant species, including rice (Mikami et al., 2015a,b), it can introduce insertion and/or deletion of a small number of bases in the target gene, thus precise genome modifications—such as base substitutions—are still difficult using CRISPR/Cas9. Base editor systems using Cas9 nickase fused to cytidine and adenosine deaminase have been developed recently; these can introduce C to T (G to A) and A to G (T to C) substitutions, respectively (Komor et al., 2016; Nishida et al., 2016; Gaudelli et al., 2017). Very recently, it was reported that C to A and C to G substitutions can be introduced by use of a new base editor consisting of Cas9 nickase fused to cytidine deaminase and glycosylase in *Escherichia coli* and human cells, respectively (Kurt et al., 2020; Zhao et al., 2020). However, the window, i.e., the possible region of base substitution, is narrow, and bystander substitution of bases adjacent to the target base occurs often. In addition, it was shown that prime editing, consisting of Cas9 nickase fused to reverse transcriptase and a prime editing guide RNA consisting of a guide RNA and RNA homologous to the target DNA, enables the introduction of small mutations, including base substitutions, in human cells (Anzalone et al., 2019). This system has been applied to rice and wheat, where it was found that not only single bases but also discontinuous, up to 4-base, substitutions could be introduced into a target gene (Lin et al., 2020; Xu et al., 2020). However, as yet, there are no reports of successful substitution of several discontinuous bases. Thus, it is not always possible to introduce the desired substitutions into a target gene using the above-mentioned systems, and the development of novel and improved GT systems is an important step toward solving this problem.

In the positive–negative-selection-mediated GT system, a positive selection marker located between both homology arms confers drug resistance to GT cells, while negative selection markers located outside the two homology arms act to kill cells in which the GT vector has integrated randomly in the genome. Desired mutations are introduced into a target site concomitant with the insertion of a positive selection marker by HR between the donor and genomic DNA, and subsequent excision of the positive selection marker from the GT locus leaves only the desired mutations (Shimatani et al., 2015). For marker excision, site-specific recombinases such as Cre-*loxP* (Sauer and Henderson, 1990), FLP-*FRT* (Golic and Lindquist, 1989), and R-RS (Onouchi et al., 1991) have been used. A marker excision system using Cre-*loxP* has been applied to removing the positive selection marker gene from the GT locus in rice (Terada et al., 2010; Dang et al., 2013). In this latter system, the “footprint,” which can be several tens of bases for recombinase recognition, remained at the target site after marker excision. In contrast, *piggyBac* transposon, derived from the cabbage looper moth, removed the selectable marker without leaving any footprint in human cells (Yusa et al., 2011; Morioka et al., 2014; Sun and Zhao, 2014). We have previously demonstrated that *piggyBac* could be applied successfully with high efficiency to remove a positive selection marker gene without leaving any footprint in rice (Nishizawa-Yokoi et al., 2015a). However, due to the *piggyBac* transposon's requirement for a TTAA sequence for transposase-dependent integration and excision, the site of the positive marker gene integration site on the GT vector must contain that motif.

Single-strand annealing (SSA) is a DSB repair system in many organisms. DNA repair by SSA occurs between homologous sequences located on both sides of the DSB site. The intervening sequences between homologous regions are eliminated by annealing single-stranded DNA of the two homologous sequences at the DSB site. Several reports have demonstrated elimination of the fragment between homologous sequences on genomic DNA via SSA attributed to DSBs in plants, including in rice (Puchta and Hohn, 1991; Kwon et al., 2012). This system had been applied to marker excision at the GT locus in mice and yeast nearly 30 years ago (Hasty et al., 1991; Valancius and Smithies, 1991). Recently, we reported precise genome editing using GT and subsequent marker excision via SSA in rice (Endo et al., 2020).

**Figure 1:**
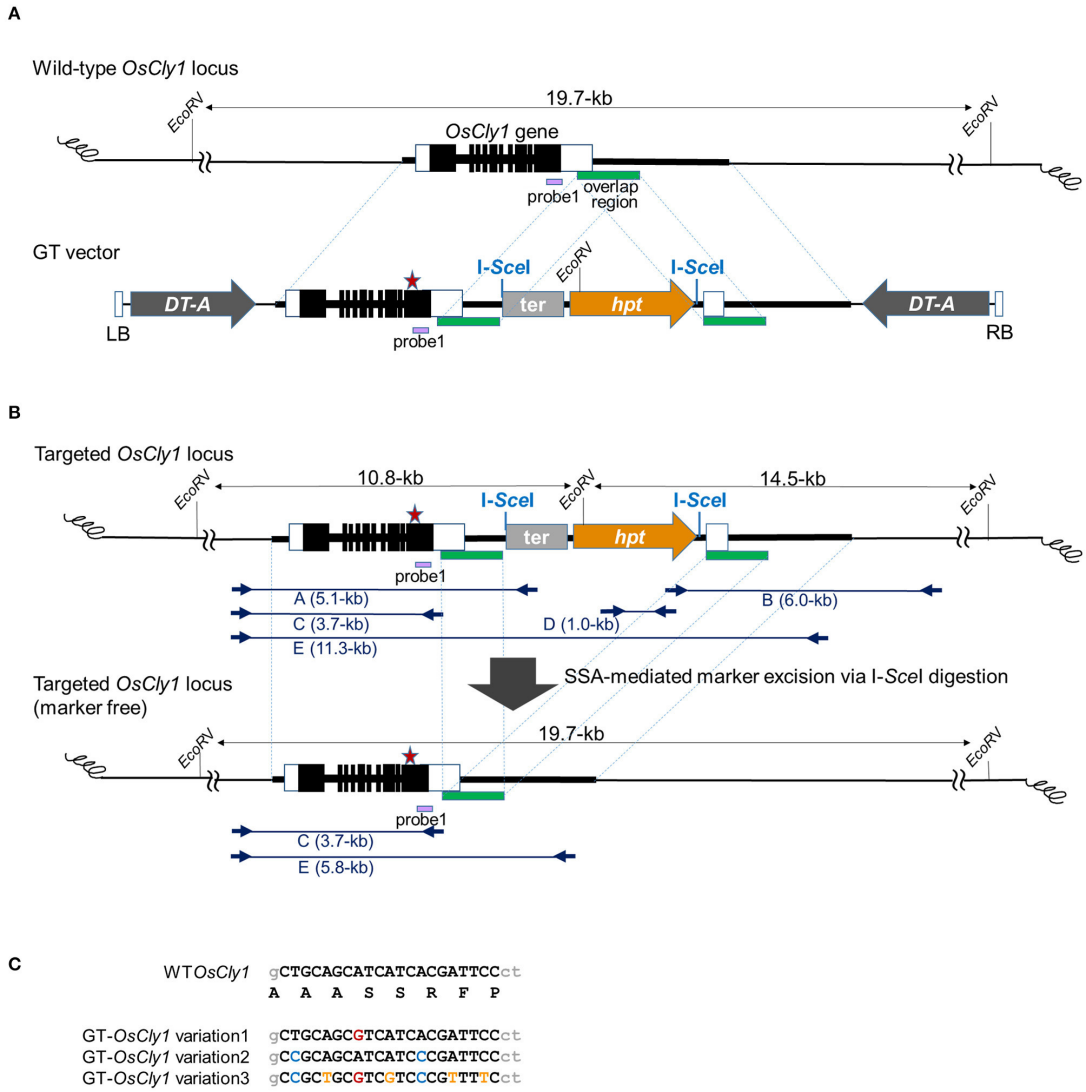
Strategy to introduce desired substitutions into the *OsCly1* gene via GT and subsequent SSA-mediated marker excision. **(A)** Schematic representation of GT at the *OsCly1* locus. The upper line indicates the wild-type (WT) *OsCly1* locus. White and black boxes indicate untranslated and coding regions of *OsCly1* gene, respectively. The lower line indicates the T-DNA region of the GT vector. The black bar indicates homologous regions of the *OsCly1* locus with 1.2-kb overlapped sequences (green bar). The red star indicates position of desired substitution(s). The GT vector carries the rice actin gene terminator (ter) and *hpt* gene under the control of the CaMV 35S promoter as a positive selection marker, and *DT-A* genes under the control of maize polyubiqutin1 promoter or rice elongation factor 1-alpha promoter as negative selection markers. The purple bar shows probe 1 for Southern blot analyses. LB and RB; left and right borders of T-DNA, respectively. **(B)** Strategy for marker excision from the GT locus using SSA. The upper line indicates the *OsCly1* locus modified by GT via HR between the GT vector shown in **(A)** and endogenous genome. The lower line indicates the GT-modified *OsCly1* locus after SSA-mediated marker excision. Blue arrows show primer sets used for PCR and sequence analyses to confirm GT and marker excision events. **(C)** Sequence alignments of desired substitution(s) in *OsCly1* gene indicated as a red star in **(A,B)**. Black uppercase letters indicate miR172 target sequences in the *OsCly1* gene. The top line indicates the genomic and deduced amino acid sequences of the WT *OsCly1* gene. The colored letters in the lower three lines indicate the substitutions in each GT vector.

Barley *cleistogamy 1* (*cly1*) has been isolated as an essential factor for cleistogamy—an unconventional pollinating style with discontinuously closed flower on some commercial cultivars—in barley (Figure 1A). *cly1* transcript levels are regulated by a microRNA (miRNA), miR172, binding at a complementary 21-bp site encoded on the 10th exon (Nair et al., 2010; Anwar et al., 2018). The *OsCly1* gene (Os04g0649100) is a homolog of barley *cly1* in rice (Zhu and Helliwell, 2011). Rice plants overexpressing the *oscly1* mutant and OsmiR172b frequently exhibit enlarged lodicules and unclosing lemma (Zhu et al., 2009; Zhou et al., 2012). These results suggest the possibility that, as in barley, miR172-mediated downregulation of *OsCly1* is involved in flower closing in rice, and that it might be possible to change opened flowering to closed flowering in rice by substitution of conserved miR172 target sequences in *OsCly1*. Moreover, we have already reported the successful introduction via GT of base substitutions at the miR172 target site in *OsCly1* and subsequent *piggyBac* transposon-mediated marker excision (Nishizawa-Yokoi et al., 2015a). Thus, the *OsCly1* gene is a suitable target gene for this study.

Here, we introduced mutations via GT then compared mutation frequencies between subsequent marker excision via either *piggyBac* transposon or SSA. In addition, we attempted to introduce not only a precise single base substitution, but also a 2-base substitution (both these 1- and 2-bp changes are found naturally among barley varieties) as well as a 7-base artificial discontinuous substitution into the miRNA target site in the *OsCly* gene.

## Materials and Methods

### Vector Construction

The GT vectors for the *OsCly* gene shown in Figure 1A were constructed as follows. To construct a vector harboring 4.9-kb of 5′ homology sequence for the *OsCly1* locus with overlapped sequences, fragments amplified by PCR using gene-specific primers (listed in Supplementary Table 1) and rice genome DNA as a template were inserted into *Asc*I/*Pme*I-digested pE(L1-L4) vector, yielding pE(L1-L4)5′OsCly1. The substitution in GT-*OsCly1* variation 1 (Figure 1C) into pE(L1-L4)5′OsCly1 was performed using a QuickChange II XL site-directed mutagenesis kit (Stratagene, USA) according to the manufacturer's protocol with the primer sets listed in Supplementary Table 1, yielding pE(L1-L4)5′OsCly1-var1. The 3.7-kb of 5′ homology sequence for *OsCly1* locus without overlapped sequences was constructed using a similar method. To construct a vector harboring 5.5-kb of 3′ homology sequence for *OsCly1* locus, fragments amplified by PCR using gene-specific primers (listed in Supplementary Table 1) and rice genome DNA were inserted into *Bam*HI/*Xho*I-digested pE(L3-L2) vector, yielding pE(L3-L2)3′OsCly1. The LR reaction for the introduction of entry vectors described above and the pE(R4-R3)I-SceITactHyg vector containing the rice actin terminator and *htp* expression cassette into the destination vector, pKOD4 (Nishizawa-Yokoi et al., 2015a) was performed using LR clonase II (Life Technologies, USA), yielding a GT vector, GT-OsCly1 variation 1. To construct GT vectors, GT-OsCly1 variation 2 and 3, 1.8-kb fragments amplified by PCR using gene-specific primers (listed in Supplementary Table 1) were replaced with GT-OsCly1 variation 1 using *Not*I/*Asc*I.

The GT vector for the *OsALS* gene was constructed as follows. To construct a vector harboring 3.4-kb of 5′ homology sequence for *OsALS* locus, pZAmGFP containing mutated OsALS harboring W548L/S627I mutations (Osakabe et al., 2005), and GFP expression cassette in a derivative of pPZP202 (Hajdukiewicz et al., 1994) was digested with *Asc*I and *Hin*dIII (blunt-ended with T4 DNA polymerase [TOYOBO, Japan]), and inserted into *Asc*I and *Pac*I (blunt-ended with T4 DNA polymerase)-digested pE(L1-L4) vector. To construct a vector harboring 6.2-kb of 3′ homology sequence for the *OsALS* locus, NBALSGT(AscI/PacI)+pPZP2028 vector (Saika et al., 2015) was digested with *Asc*I and *Pac*I, and inserted into *Asc*I/*Pac*I-digested pE(L3-L2) vector. To construct a positive selection marker, pCAMBIA1390-sGFP (Toki et al., 2006) was digested with *Xba*I and *Pst*I (blunt-ended with T4 DNA polymerase). The resulting 0.7-kb fragment of GFP was inserted into *Xba*I and *Bam*HI (blunt-ended with T4 DNA polymerase)-digested pE(R4-R3)I-SceITactHygcodA vector containing the rice actin terminator and *htp* expression cassette. The LR reaction for the introduction of the three vectors described above into the destination vector, pKOD4 (Nishizawa-Yokoi et al., 2015a) was performed using LR clonase II (Life Technologies).

The I-*Sce*I expression vector shown in Figure 3A was constructed by LR reaction for introduction of the following vectors into the destination vector pZD202 (Kwon et al., 2012), pE(L1-L4)Pg3pDsRed2Tg3p containing a 2.4-kb fragment of the rice glyceraldehyde-3-phosphate (G3P) promoter, a 0.8-kb fragment of intron-DsRed and a 0.8-kb fragment of the rice G3P terminator, pE(R4-R3)TactP35SnptIIThsp (Nishizawa-Yokoi et al., 2015b) and pE(L3-L2)P2X35S::I-SceI::Thsp (Kwon et al., 2012). For construction of the control vector, pE(L3-L2)T17.3 containing a 1.2-kb fragment of rice heatshock protein 17.3 terminator was used for the LR reaction instead of pE(L3-L2)P2X35S::I-SceI::Thsp.

### *Agrobacterium*-Mediated Transformation

GT vectors were transformed into *Agrobacterium tumefaciens* strain EHA105 (Hood et al., 1993) by the electroporation method as shown schematically in Supplementary Figure 1. Rice (*Oryza sativa*. L cv. Nipponbare) was used for *Agrobacterium*-mediated transformation as described previously (Toki, 1997; Toki et al., 2006). Briefly, 3-week-old secondary calli transformed with *Agrobacterium* harboring pKOD4/OsCly1 were selected on N6D medium solidified with 0.4% gelrite containing 50 mg/L hygromycin and 25 mg/L meropenem. GT candidate calli confirmed as below were transferred to regeneration medium with 25 mg/L meropenem, and shoots arising from callus were transferred to MS medium without phytohormones. For marker excision, GT calli in the T_0_ generation or induced from mature seeds in the T_1_ generation were transformed with *Agrobacterium* harboring the I-*Sce*I expression vector. Transformed calli were selected on N6D medium containing 35 mg/L G418 (Geneticin) and 25 mg/L meropenem. Marker-free calli confirmed as below were transferred to regeneration medium.

### Screening of GT and Marker Excision Events

Genomic DNA was extracted from hygromycin resistant calli after 4–5 weeks selection and from leaves of regenerated plants by Agencourt Chloropure (Bechman Coulter, USA) according to the manufacturer's protocol. PCR analysis was performed with PrimeSTAR GXL DNA Polymerase (TAKARA BIO) or KOD FX neo (TOYOBO, Japan) using the primer sets listed in Supplementary Table 1. For direct sequence analysis, amplified fragments were purified with a QIAquick Gel Extraction Kit (Qiagen, Germany). Sequences of purified PCR fragments were read with an ABI3130 sequencer (ABI, USA) and analyzed with Sequence Scanner.

### Southern Blot Analysis

Genomic DNA was extracted from leaves of GT candidate plants using a Nucleon Phytopure Extraction Kit (GE Healthcare, USA) according to the manufacturer's protocol. Genomic DNA (2 μg) was digested with *Eco*RV or *Msc*I and gel electrophoresis performed in a 0.8% gel with around 30 V. Specific DNA probes were prepared using a PCR digoxigenin (DIG) probe synthesis kit (Roche Diagnostics, Switzerland) according to the manufacturer's protocol using the primer sets listed in Supplementary Table 1. Southern blot analyses were performed by following a conventional protocol.

### Observation of Floral Tissues by Optical Microscopy

GT homozygous plants in the T_1_ and T_2_ generations, GT#34-6-53 and #441-113-115-38, respectively, were grown in a greenhouse. Floral tissues of 2.5-month-old plants were observed with a microscope (LEICA DFC310 FX, Germany) as previously described (Yoshida et al., 2007; Lombardo et al., 2017).

### Observation of GFP and DsRed Fluorescence

GFP and DsRed fluorescence from rice calli was observed using a fluorescence microscope with GFP2 and DsRed filters, respectively (MZ FLIII).

## Results and Discussion

### Precise Modification of the miR172 Target Site in the *OsCly1* Gene via Positive–Negative Selection-Mediated GT

The T-DNA structures in GT vectors used in this study are illustrated in Figure 1A. In the GT vectors, endogenous rice genomic sequence from the *OsCly1* locus with desirable substitutions at the miR172 target site (GT-*OsCly1* variation 1, 2, and 3 in Figure 1C) was interrupted by the positive selection marker consisting of the cauliflower mosaic virus (CaMV) 35S promoter, *hygromycin phosphotransferase* (*hpt*) gene, and rice actin gene terminator. The purpose of the rice actin terminator was to help prevent transcriptional drive-through from the *OsCly1* gene to the downstream *hpt* gene. I-*Sce*I meganuclease recognition sequences were placed at both ends of the *hpt* selection marker cassette. Partially overlapped sequence of the *OsCly1* gene of 1.2-kb in length was located at the 3′ end of the *hpt* cassette to induce break-induced SSA for excision of the *hpt* cassette. *Diphtheria toxin A subunit* (*DT-A*) gene expression cassettes as a negative selection marker were located just inside the left and right borders to suppress growth of hygromycin-resistant cells in which the GT vector is integrated randomly into the rice genome.

**Figure 2:**
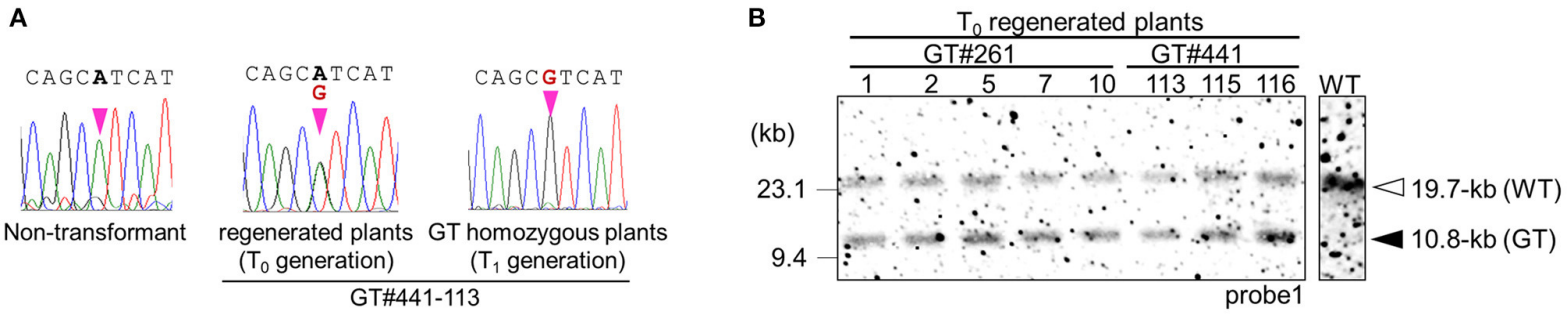
Molecular analyses confirming GT events in GT#261 and GT#441. **(A)** Sequencing chromatograms at the mutation site on the *OsCly1* gene in a non-transformed plant, and in GT plants from the T_0_ and T_1_ generations. **(B)** Southern blot analyses with probe1 (Figure 1) using *Eco*RV-digested genomic DNA of regenerated plants from GT calli of GT#261 and GT#441. White and black triangles indicated bands derived from WT and GT allele, respectively.

First, we performed GT experiments using the vector GT-OsCly1 variation 1 to introduce the single base substitution found in the *cly1* gene of cleistogamous barley varieties, which is the same substitution as our previous report (Nishizawa-Yokoi et al., 2015a). The A to G substitution in GT-*OsCly1* variation 1 is located at the 8th position of the miRNA172 target sequence in the *OsCly1* gene (Nair et al., 2010). Rice calli transformed with GT-OsCly1 variation 1 were cultured on medium containing hygromycin B for 4 weeks. A total of 1,476 hygromycin-resistant calli were obtained from 8,239 (~56 g) pieces of *Agrobacterium*-infected calli (Table 1). To screen GT calli, PCR analyses with primer sets A and B to amplify 5′ and 3′ regions of the targeted locus shown in Figure 1B were performed. Both 5′ and 3′ junction fragments were detected in a total of 30 independent lines (Table 1), indicating that the *hpt* gene was introduced into the *OsCly1* locus by HR between the GT vector and endogenous target sequences. Regenerated plants from these 30 lines of GT-positive calli were analyzed. Direct sequence analyses of PCR fragments amplified with primer set C showed that a heterozygous base substitution A/G, at the 8th position of the miRNA172 target site in the *OsCly1* gene, was found in eight lines of regenerated plants (Figure 2A), suggesting that true GT events had occurred in these plants. Southern blot analyses of *Eco*RV-digested DNA from these eight lines, using probe 1 recognizing the endogenous *OsCly1* gene (Figure 1), showed that wild-type (WT) bands (19.7-kb) and bands corresponding to the GT allele (10.8-kb) were detected in five lines of regenerated plants, although only 19.7-kb bands were detected in non-transformed plants (Figure 2B). Taken together, these molecular analyses showed that precise introduction of the *hpt* gene and desired substitution into the *OsCly1* gene via GT had occurred successfully in a total of five independent plants.

**Table 1:** Summary of GT experiments for *OsCly1* locus.

**GT vector**	**Substitution (same as Figure 1C)**	**Overlapped region**	**No. of** ***Agrobacterium*****-infected** **calli**		**No. of hygromycin-resistant calli**	**No. of targeted!!break calli**	**No. of targeted calli with desired mutations**
GT-*OsCly1* variation 1	CTGCAGCGTCATCACGATTCC	No overlap	2,069 (46.69 g)		179	5	2
		1.2-kb of overlapped sequence	Exp.1	2,336 (22.64 g)	495	10	2
			Exp.2	2,658 (15.11 g)	645	10	2
			Exp.3	3,245 (18.31 g)	336	10	1
			Total	8,239 (56.06 g)	1476	30	5
GT-*OsCly1* variation 2	CCGCAGCATCATCCCGATTCC	1.2-kb of overlapped sequence	1404 (9.56 g)		107	6	3
GT-*OsCly1* variation 3	CCGCTGCGTCGTCCCGTTTTC	1.2-kb of overlapped sequence	1560 (11.33 g)		120	2	1

### Effect on GT Efficiency of Overlapping Sequence in the GT Vector

We expected that the *hpt* marker cassette would be excised from the GT locus by I-*Sce*I-dependent break-induced SSA using the 1.2-kb overlapped sequence (Figure 1B). We previously reported that SSA occurs spontaneously in rice, although at low frequency (Kwon et al., 2012). Thus, the marker cassette could be removed from the GT vector or GT locus in the absence of I-*Sce*I through spontaneously induced DSBs and subsequent DSB repair by SSA. If the *hpt* marker cassette is removed before GT, GT candidate cells cannot be selected with hygromycin. Similarly, if the *hpt* marker cassette is removed from the GT locus, GT cells cannot survive on medium containing hygromycin. To assess whether spontaneously occurring SSA could decrease the efficiency of GT cell selection, we compared the frequencies with which GT lines were obtained between GT-OsCly1 variation 1 vectors without overlapped sequences, as shown in Supplementary Figure 2A. GT experiments and molecular analyses of calli and regenerated plants showed that two lines of true GT regenerated plants (#34 and 62) were obtained from 2,069 pieces of *Agrobacterium*-infected calli (Table 1; Supplementary Figures 2B,C). Moreover, in our previous study, it was shown that two GT calli carrying an A/G mutation in the miR172 targeting site of the *OsCly1* gene were obtained from 5,139 pieces of *Agrobacterium*-infected calli (Nishizawa-Yokoi et al., 2015a). These results showed that the frequency, i.e., the ratio of the number of GT lines to that of *Agrobacterium*-infected calli using a GT vector without overlapping sequences, was estimated as 0.1%, which is not greatly different from that using a GT vector with overlapping sequences (0.06%) (Table 1). Moreover, in this experiment, the GT frequency, i.e., the ratio of GT cells to transformed cells (e.g., hygromycin-resistant cells), is estimated as 2.8 and 2.3% using a GT vector with and without overlapping sequences, respectively, which is similar to that reported previously (generally 0.1–10%; Shimatani et al., 2015). These results suggest that spontaneous SSA-mediated marker excision occurred only rarely in our experiments. We have recently reported a successful example of GT- and SSA-mediated marker excision using 30-bp overlapped sequences in a GT vector (Endo et al., 2020). Here, the frequency (as defined above) was similar between GT vectors with/without 1.2-kb overlapped sequences, suggesting that the use of short overlapped sequences may not be necessary in this experiment.

One of the difficulties of performing GT in higher plants is its very low frequency, due mainly to the low HR frequency. In rice, GT cells via naturally occurring HR can be screened if not using a sequence-specific nuclease such as CRISPR/Cas9. Just recently, we reported a CRISPR/Cas9-mediated DNA DSB-induced GT system using a vector harboring a CRISPR/Cas9 expression construct, selectable marker, and GT donor template (Nishizawa-Yokoi et al., 2020). Moreover, our previous report showed that DSB induction via CRISPR/Cas9, in combination with a deficiency of Ligase 4—a key enzyme in NHEJ competing with HR—could enhance GT frequency in rice (Endo et al., 2016). DSB induction via CRISPR/Cas9 will be used to improve positive–negative selection-mediated GT frequency in this experiment also.

### Precise Elimination of a Positive-Marker Cassette From the GT Locus via I-*Sce*I-Mediated Break-Induced SSA

**Figure 3:**
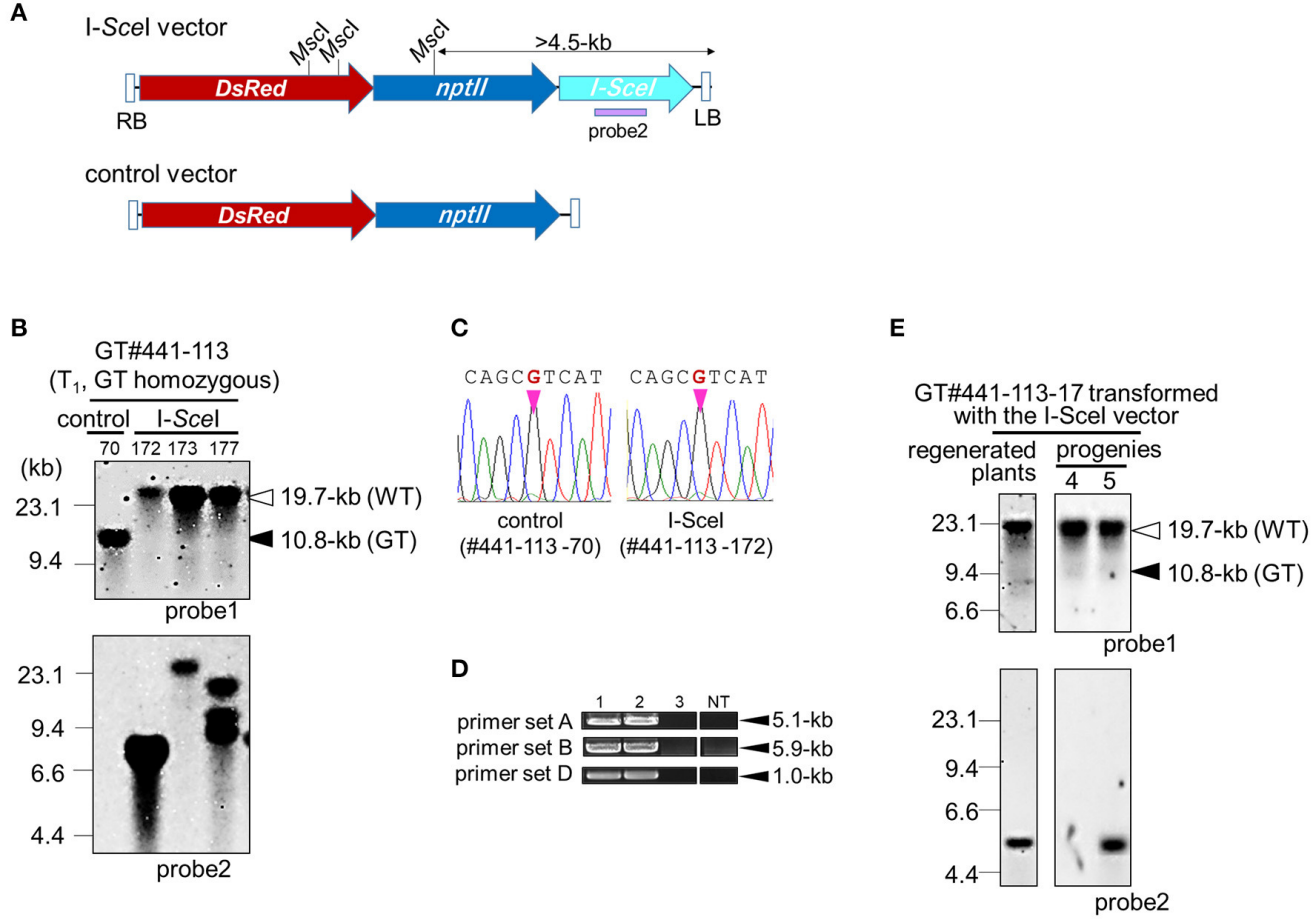
Marker excision from GT locus in GT#441. **(A)** Schematic diagrams of T-DNA for the I-*Sce*I expression vector. The I-SceI vector (top) carries the I-*Sce*I gene under the control of CaMV 35S promoter, *npt* gene, and *DsRed* gene. The control vector (bottom) carries no I-*Sce*I expression cassette. A purple bar indicates probe 2 for Southern blot analysis. **(B)** Southern blot analyses with probe 1 or 2 using *Eco*RV or *Msc*I-digested genomic DNA, respectively. Samples are GT homozygous plants in the T_1_ generation transformed with the I-SceI vector or control vector. White and black triangles indicated bands derived from the WT and marker-excised GT allele, and the GT allele, respectively. **(C)** Sequencing chromatograms at the mutation site on the *OsCly1* gene in GT#441-113-70 and GT#441-113-172. **(D)** PCR analysis to confirm marker excision. Sample 1: regenerated plants of GT#441-113; samples 2 and 3: GT homozygous plants of GT#441-113-70 and GT#441-113-172 transformed with control and I-SceI vector, respectively. NT; non-transformant. **(E)** Southern blot analyses with probe 1 or 2 using *Eco*RV- or *Msc*I-digested genomic DNA, respectively. Samples are regenerated plants and the progenies of GT homozygous plants of GT#441-113-17 (T_1_ generation) transformed with the I-SceI vector. Details as in **(B)**.

As the *hpt* gene is no longer needed after selection of true GT cells, the *hpt* gene cassette was excised from the GT locus by I-*Sce*I-mediated break-induced SSA. Here, two lines, GT#261 and GT#441 (Figure 2), were used for marker excision experiments, as shown schematically in Figure 1B and Supplementary Figure 1. Homozygous or heterozygous GT callus lines derived from T_1_ seeds of GT#261 and GT#441 were infected individually with *Agrobacterium* harboring an I-*Sce*I expression vector driven by a double CaMV 35S promoter (Kwon et al., 2012) as shown in Figure 3A. *Agrobacterium*-infected calli were selected on medium containing G418. To screen cells in which the positive selection marker had been excised successfully from the GT locus in G418-resistant calli, PCR analysis with primer sets B and E (Figure 1B) was performed. Primer set B amplifies a 6.0-kb band in GT lines if the positive selection marker remains in the *OsCly1* locus, but not in marker-excised lines; primer set E amplifies a 11.3-kb band in GT lines still containing the positive selection marker in the *OsCly1* locus, while a 5.8-kb band is amplified in marker-excised lines and WT. As summarized in Table 2, the positive selection marker was excised from the *OsCly1* locus in over 25 and 90% of calli heterozygous and homozygous for the GT allele, respectively. Interestingly, bi-allelic marker excision was detected in 38 and 20% of calli in GT#261 and GT#441, respectively. In contrast, marker excision was not found in calli transformed with a control vector lacking the I-*Sce*I expression construct. In general, G418-resistant callus is a mosaic of marker excised and non-excised cells; thus, PCR fragments could be amplified using primer set B from cells neighboring those in which a positive marker was excised. Consequently, PCR analysis might underestimate marker excision frequency.

**Table 2:** Frequency of SSA-mediated marker excision from *OsCly1* locus.

**GT Vector**	**GT calli lines**		**Vector**	**Marker excision from** ***OsCly1*** **locus**			
	**Line No. (generation)**	**GT allele**		**Without marker**		**With marker**	**Total**
				**Mono-allelic**	**Bi-allelic**		
GT-*OsCly1* variation 1	GT#441-113 (T_1_)	homozygous	Control	0	0	16	16
			I-*Sce*I	18	12	2	32
		heterozygous	Control	0	-	15	16
			I-*Sce*I	18	-	14	32
	GT# 261-7 (T_1_)	homozygous	Control	0	0	16	16
			I-*Sce*I	22	6	2	30
		heterozygous	Control	0	-	16	16
			I-*Sce*I	8	-	24	32
		heterozygous	Control	0	-	16	16
			I-*Sce*I	5	-	11	16
	GT# 137 (T_0_)	homozygous	Control	0	-	11	11
			I-SceI	18	-	25	43

Regenerated plants were obtained from homozygous GT calli in which the positive selection marker had been excised successfully from the GT locus. In GT#441, Southern blot analysis of *Eco*RV-digested DNA from GT homozygous plants with probe 1 revealed a 19.7-kb band in both WT and a marker-excised GT line transformed with the I-*Sce*I vector, although bands for the GT allele (10.8-kb) were detected in regenerated plants transformed with a control vector (Figure 3B). This result suggests that the positive selection marker was completely excised from mono-allelic or bi-allelic *OsCly1* loci as expected. Direct sequence analyses of PCR fragments amplified with primer set E revealed that the desired mutations found in calli were maintained in these plants (Figure 3C). Moreover, PCR analyses of plants of GT#441-172 and GT#441-85 confirmed marker excision. Primer sets A, B, and D amplify fragments in case of successful targeted integration of the positive selection marker in *OsCly1* gene but not in WT or marker-excised lines (Figure 1B). As expected, fragments were not amplified in these lines using these primer sets (Figure 3D). In addition, Southern blot analysis using probe 2 revealed that the copy number of the I-*Sce*I vector was low in these plants (Figures 3B,E). In the next generation, line #441-113-17, in which a single copy of T-DNA was integrated, T-DNA of the I-*Sce*I vector inserted into the rice genome was segregated, and marker-excised plants without the I-*Sce*I vector were obtained successfully in both lines (Figures 3B,E). We confirmed successful marker excision in GT#261 also (Supplementary Figure 3). Thus, following marker excision, plants harboring precise genome editing with the desired point mutation in the miR172 target site in the *OsCly1* gene were obtained successfully by a combination of a positive–negative selection-mediated GT approach and subsequent SSA-mediated precise excision of the positive selectable marker.

### Marker Excision Before Regeneration in the GT T_0_ Generation

**Figure 4:**
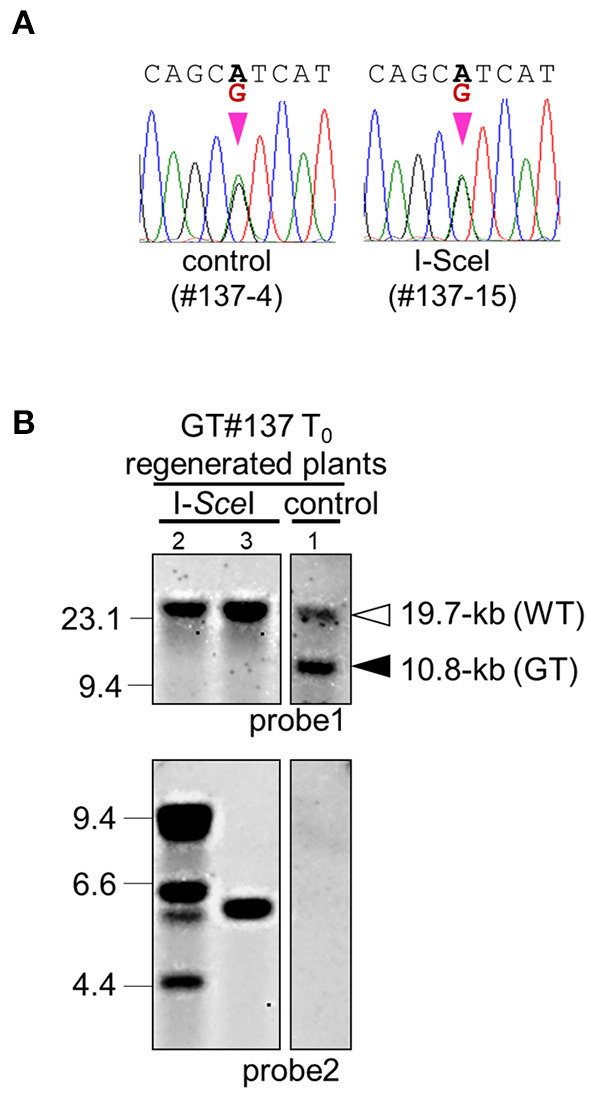
Molecular analysis to confirm GT and marker excision events in GT#137. **(A)** Sequencing chromatograms of regenerated plants GT#137-4 and GT#137-15 transformed with control and I-*Sce*I vector, respectively. Sequences were determined using fragments amplified with primer set **(C)** in Figure 1B. **(B)** Southern blot analyses with probe 1 or 2 using *Eco*RV or *Msc*I-digested genomic DNA, respectively. Samples are regenerated plants of GT#137 transformed with the I-*Sce*I vector or control vector. Details as in Figure 3B.

Next, to shorten the total experimental time, we attempted to excise the positive marker gene cassette immediately after GT. The T_0_ callus lines GT#137 and GT#441, confirmed as GT events by PCR and Southern blot analysis, were used in this experiment (Exp. 3 in Table 1; Supplementary Figure 1). Three months after the first transformation of the GT vector, calli were infected with *Agrobacterium* harboring the I-*Sce*I vector (Figure 3A). One and half months after onset of G418 selection, excision of the positive selection marker was confirmed by PCR analyses with primer pair A or B. A total of 18 calli from 43 I-*Sce*I-transformed calli were seen to have lost the positive marker gene, whereas there were no marker-free calli in 11 lines transformed with a control vector (Table 2). Several plants regenerated from those GT#137 T_0_ calli were analyzed further. Direct sequencing of PCR fragments amplified using primer set C or E showed the simultaneous detection of superposing signals of A and G at the 8th positions at the miRNA172 target site in the *OsCly1* gene in plants transformed with the I-*Sce*I vector (Figures 4A,B; Supplementary Figure 4). The results of Southern blot analyses of *Eco*RV- or *Msc*I- digested genomic DNA with probe 1 or 2, respectively, also supported the loss of the positive selection marker from the GT locus accompanying I-*Sce*I expression in this generation (Figure 4B; Supplementary Figure 4B). Thus, we had again successfully introduced the desired substitutions into the rice acetolactate synthase (*OsALS*) gene by GT and SSA-mediated marker excision (Supplementary Figure 5; Supplementary Table 2).

Notably, the SSA-mediated repair system does not have any limitations regarding the donor sequence on the GT vector, whereas the *piggyBac* system needs the “TTAA” recognition sequence to allow transposase PBase to remove the positive marker (Nishizawa-Yokoi et al., 2015a). Thus, for some specific genomic regions, application of the *piggyBac* system would be troublesome. On the other hand, we found that the efficiency of SSA-mediated marker excision was lower than that of the *piggyBac*-mediated system: in T_0_ calli, the efficiency of the *piggyBac*-mediated system was nearly 100%, while that of the SSA-mediated system was around 40% (Table 2). The latter is thought to be adequate to produce marker-free rice plants. However, marker excision frequency would need to be further improved for plant species in which it is difficult to separate marker-excised cells from a mosaic of marker-excised and non-excised cells. It has been reported that SSA is not the sole DNA repair pathway in rice and Arabidopsis, even if overlapped sequences surround the DSB site (Kwon et al., 2012; Vu et al., 2014). Marker excision frequency might be improved by enhancement of the SSA pathway or suppression of DSB repair pathways other than SSA. We previously demonstrated that SSA can be enhanced by overexpression of rice exonuclease, OsExo1 and/or OsRecQl4 helicase (Kwon et al., 2012). Thus, overexpression of OsExo1 and OsRecQl4 would be expected to improve SSA-mediated marker excision.

### Introduction of Multiple Substitutions in the *OsCly1* Gene

It is expected that the lower homology between the miR172 sequence and its target sequence in the *OsCly1* gene would result in greater tolerance to miR172-mediated downregulation of *OsCly1*. Therefore, we attempted to introduce multiple substitutions at the miR172 target site in the *OsCly1* gene. Another two GT vectors designed to introduce multiple substitutions at the miR172 target site were constructed (Figure 1C). GT-OsCly1 variation 2 also mimicked natural variations in the *cly1* gene of cleistogamous barley varieties. The substitutions in GT-*OsCly1* variation 2, T to C and A to C, are located at the 2nd and 14th positions of the miRNA172 target site in the *OsCly1* gene, respectively (Nair et al., 2010). On the other hand, GT-*OsCly1* variation 3 harbors not only three substitutions, located at the 2nd, 8th, and 14th positions at miRNA172 target site found as natural variations in barley, but also four artificial substitutions at all the triplet codon 3rd positions of the *OsCly1* gene. In designing the four artificial substitutions, care was taken not to create “rare codons.” It is expected these substitutions will only affect transcript levels regulated by miRNA172 target because there are no base substitutions altering amino acid residues of the OsCly1 protein.

**Figure 5:**
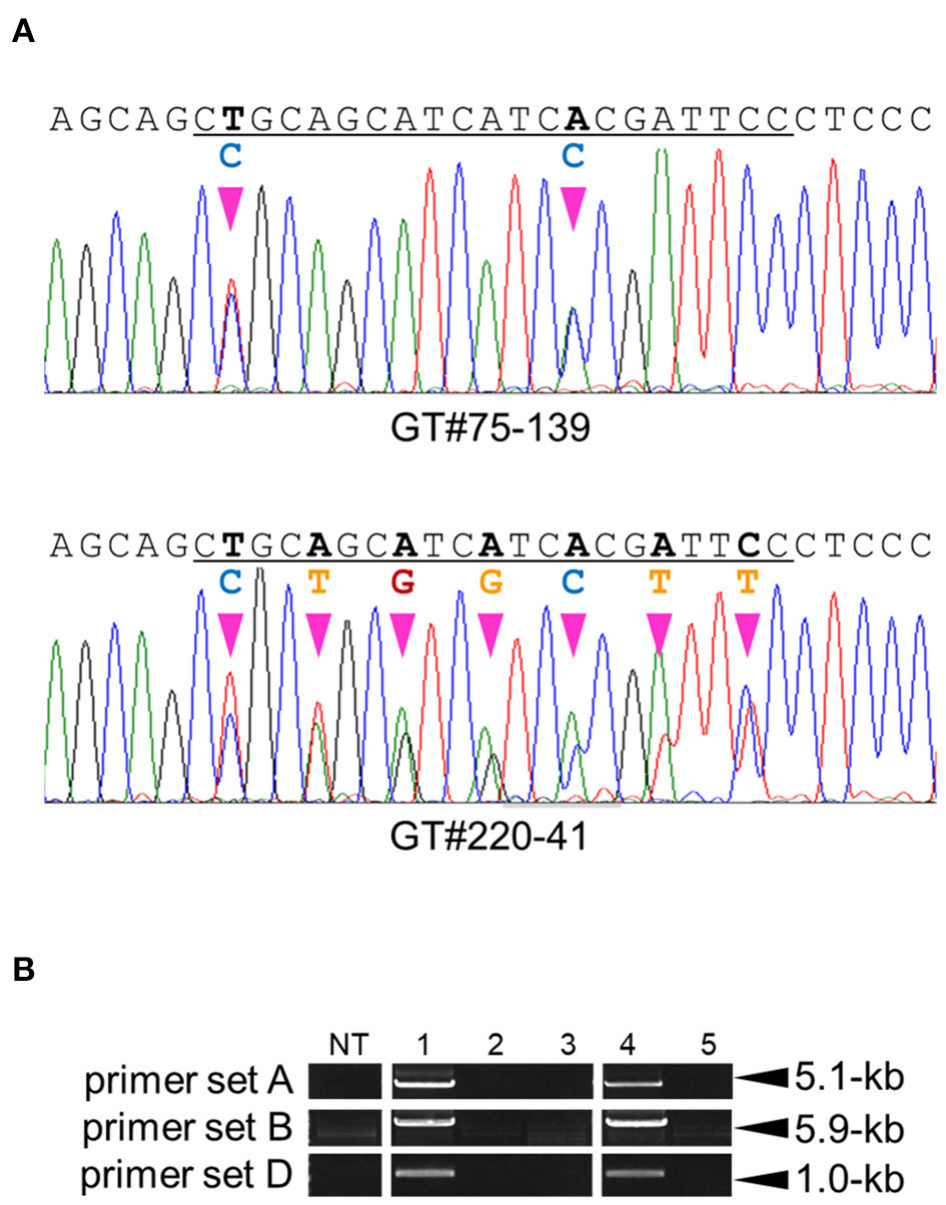
Molecular analysis to confirm GT and marker excision events in GT#75 and 220. **(A)** Sequencing chromatograms of regenerated plants, GT#75-139 and GT#220-41 derived from GT calli transformed with GT vectors harboring mutations induced by GT-OsCly1 variation 2 and 3, respectively. The miR172 target site region is underlined. **(B)** PCR analysis to confirm marker excision in GT#75 and GT#220. Samples 1–3: GT regenerated plants (GT#75) transformed with control vector (1), and I-*Sce*I vector (2, 3). Samples 4, 5: GT regenerated plants (GT#220) transformed with control vector (4), and I-*Sce*I vector (5). NT; non-transformant. Details as in Figure 3D.

GT experiments using vectors GT-*OsCly1* variation 2 and 3 were performed as described above. Finally, 3 and 1 true independent GT lines with the desired substitutions were obtained from 1,404 and 1,560 calli transformed with GT-OsCly1 variation 2 and 3 vectors, respectively (Table 1). GT frequencies using GT-*OsCly1* variation 2 and 3 were thought to be comparable to those using GT-*OsCly1* variation 1 (Table 1). To confirm precise genome editing in regenerated plants obtained from GT calli, molecular analyses were performed in GT#75 and GT#220 in GT-*OsCly1* variations 2 and 3, respectively. Direct sequence analysis revealed that desired substitutions of 2 and 7 bases at the miR172 target site in the *OsCly1* gene were introduced successfully in T_0_ plants (Figure 5A). PCR analysis also showed successful marker excision from the GT allele in regenerated plants (Figure 5B). In sum, we demonstrate that our system could be used for precise rice genome modifications, from single base substitutions to multiple discontinuous base changes.

### Phenotype of *OsCly1*-Edited Rice Plants

**Figure 6:**
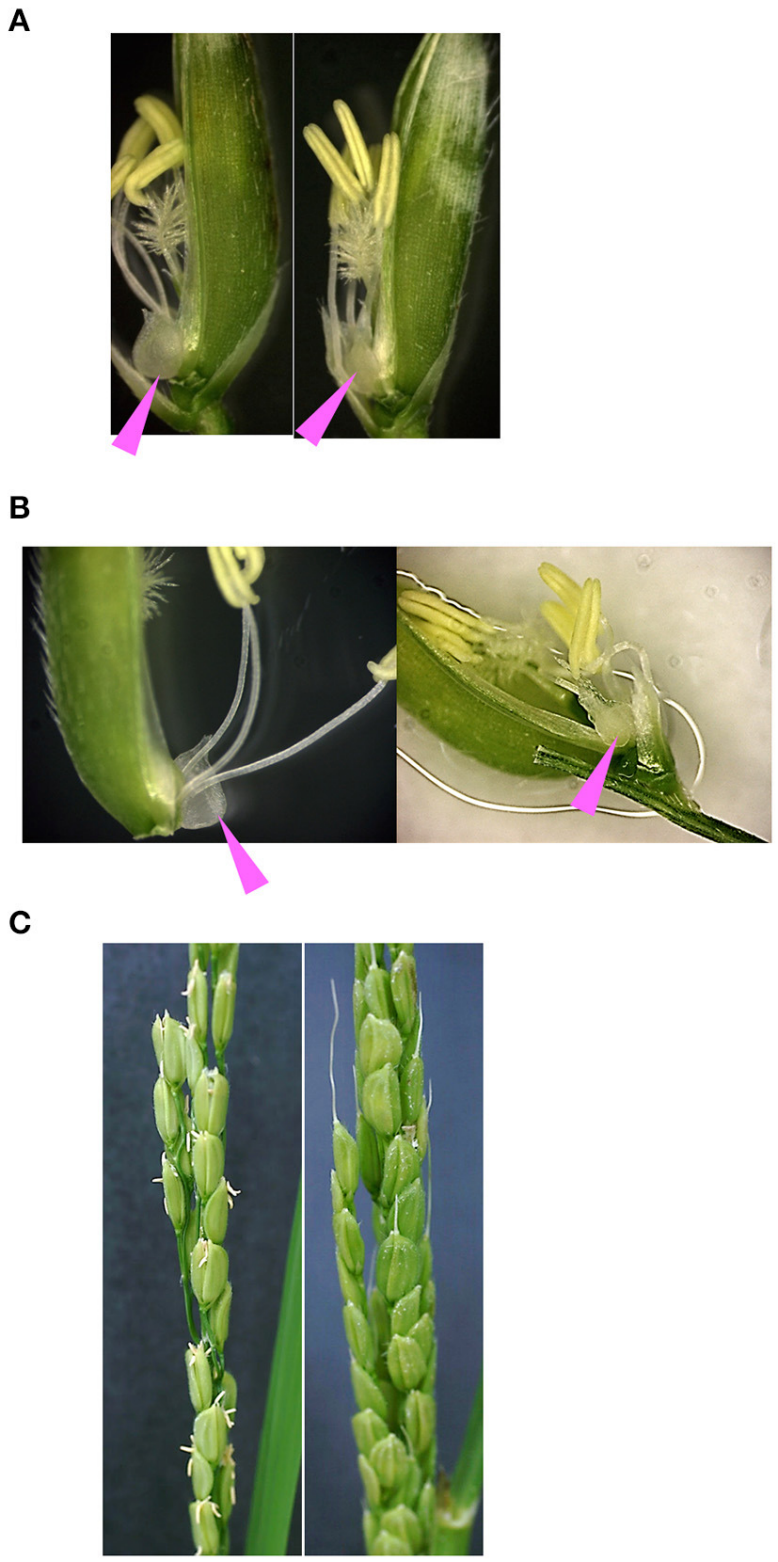
Phenotype of floral organs in GT homozygous plants. Photos of segregated WT (left) and GT homozygous (right) plants in the T_1_ generation harboring an A to G substitution at the 8th position (variation 1) at the miR172 target site in the *OsCly1* gene possessing a positive selection marker. Pink arrowheads indicate lodicules. **(A,B)** Spikelets (WT and GT plants) of #34-6-53 observed under a light microscope. **(C)** Panicles (WT and GT plants) of #441-113-115-38 at flowering time.

In barley, variations in miR172 target sequences in the *cly1* gene are involved in the cleistogamous phenotype (Nair et al., 2010). In addition, the rice *oscly1* mutant frequently showed enlarged lodicules (Zhou et al., 2012). Here, we observed the floral organs in GT homozygous plants with A to G substitution at the 8th position at miRNA172 target sequences in *OsCly1* gene (GT-*OsCly1* variation 1, Figure 1C). We grew GT homozygous plants harboring the positive selection marker, GT#34-6-53 and #441-113-115-38, in a greenhouse under natural long-day conditions. In GT#34-6-53 (see Supplementary Figure 2C, T_1_ generation), the size of lodicules in GT plants was significantly smaller than that of WT lodicules (Figure 6A), similar to the phenotype observed in the recessive *cly1* homozygous barley plants (Nair et al., 2010). Furthermore, GT plants showed much less stamen exertion compared with WT plants (Figure 6B). Interestingly, closed flowers were observed in #441-113-115-38 (a progeny of #441-113 in Figure 2B, T_2_ generation), although flowers opened in segregated WT (Figure 6C), suggesting that sequence variation (variation 1) at the miR172 target site in the *OsCly1* gene would be involved in cleistogamous flowering, as in the case of barley *cly1*. Further observation of GT plants without a positive selection marker and with other substitutions (GT-*OsCly1* variation 2 and 3) is planned in the near future.

### Future Prospects

Here, we demonstrated that GT and SSA-mediated marker excision allows desired mutations such as substitution of 2 and 7 discontinuous bases to be introduced into a target gene in rice. An earlier report showed that 18 single-base substitutions and 3 single-base deletions were introduced simultaneously at sites within 12.2-kb target sequences in rice via positive–negative-mediated GT (Johzuka-Hisatomi et al., 2008). As discussed in the *Introduction*, this GT and marker excision system could be a powerful tool to precisely modify target sequences that are difficult to access using conventional mutagenesis, base editors and prime editing. To induce SSA, we transformed GT calli with an I-*Sce*I expression vector. This might cause more somaclonal mutations, with resultant reduced regeneration ability, due to the nature of long de-differentiated callus culture. Inducible I-SceI expression is a possible approach to prevent this problem, although strict ON/OFF regulation of I-SceI expression would be necessary. Moreover, we succeeded in producing rice plants with the desired phenotype via precise mutagenesis of the miRNA target site in the *OsCly1* gene. miRNAs regulate important agronomical traits such as grain number, filling rate, fertility, and leaf inclination in rice (Peng et al., 2019). For example, a single substitution in the miR156 target site of *OsSPL14* gene involves *OsSPL14* mRNA level regulated by miR156, resulting in an increase in grain yield (Jiao et al., 2010; Miura et al., 2010). Substitutions to inhibit miRNA binding to its target gene via the system presented in this study could produce valuable rice plants. Moreover, in general, there are homologs that show highly conserved sequences in some miRNAs (Reinhart et al., 2002). Precise modification by our system enables the expression levels of miRNA-targeted genes to be regulated more strictly and specifically.

## Data Availability Statement

The original contributions presented in the study are included in the article/Supplementary Materials, further inquiries can be directed to the corresponding authors.

## Author Contributions

TK, KH, ST, and HS designed the experiments. NO, KK, AM, AN-Y, HY, and HS performed the experiments. NO and HS wrote the article with contributions of all the authors. ST supervised and complemented the writing.

## Conflict of Interest

ST has filed a patent application related to this work. Authors KK and KH were employed by the company, Nisshin Flour Milling Inc. The remaining authors declare that the research was conducted in the absence of any commercial or financial relationships that could be construed as a potential conflict of interest.
