# Supplementary material for: Precise Genome Editing in miRNA Target Site via Gene Targeting and Subsequent Single-Strand-Annealing-Mediated Excision of the Marker Gene in Plants
Source: Front Genome Ed. 2021 Jan 12;2:617713. doi: 10.3389/fgeed.2020.617713 (PMC8525353; doi:10.3389/fgeed.2020.617713)
Supplement: Supplementary file 1 [file Data_Sheet_1.PDF]

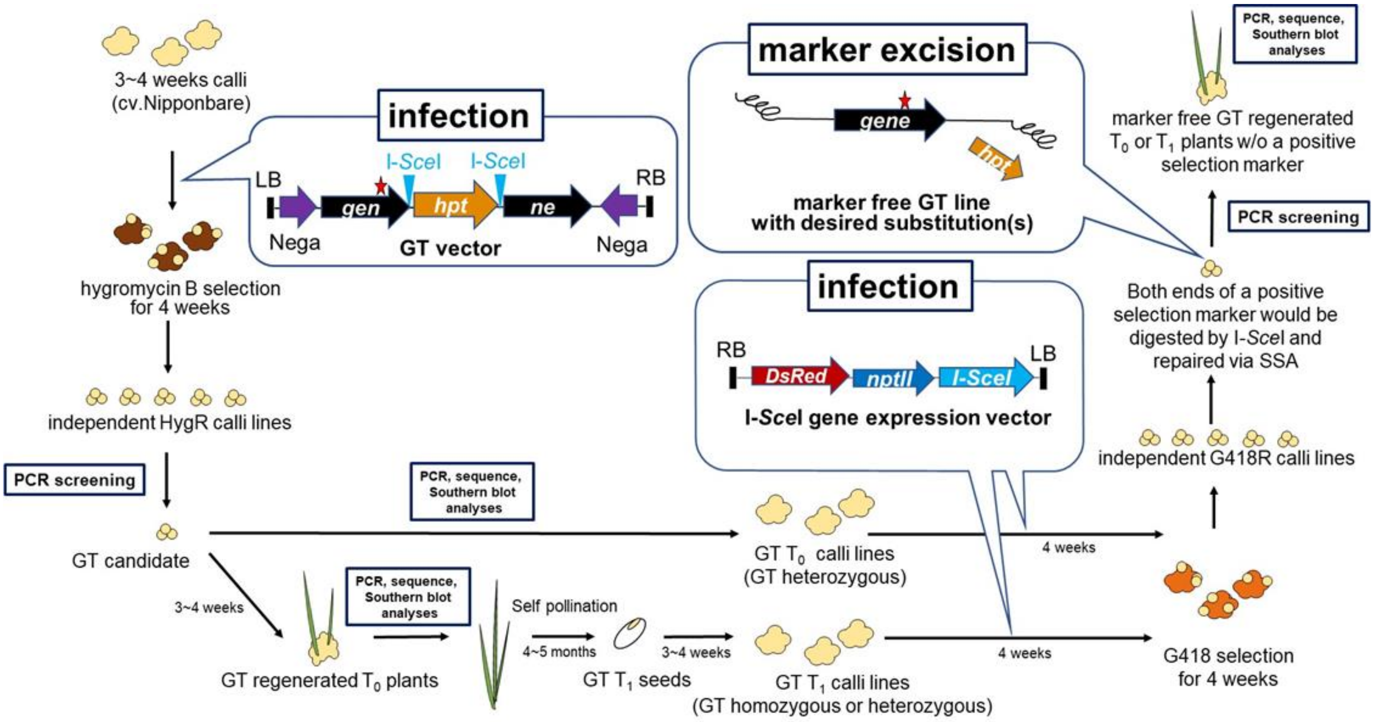

Supplementary Figure S1. Experimental flowchart of precise genome editing by a combination of GT via positive-negative selection with subsequent SSA-mediated marker excision.

Three-week-old rice calli derived from mature seeds (cv. Nipponbare) were infected with *Agrobacterium* harboring GT vector to introduce desired substitution(s) into the *OsCly1* gene (Figure 1A and C) and selected on the medium containing hygromycin. Molecular analyses (PCR, direct sequence and Southern blot analyses) were performed to screen true GT events in GT candidate calli and regenerated plants. GT calli in T_0 or T_1 generations were again infected with *Agrobacterium* harboring the I-SceI vector (Figure 3A). Calli were selected with G418 and marker excision events were confirmed by PCR and Southern blot analyses.

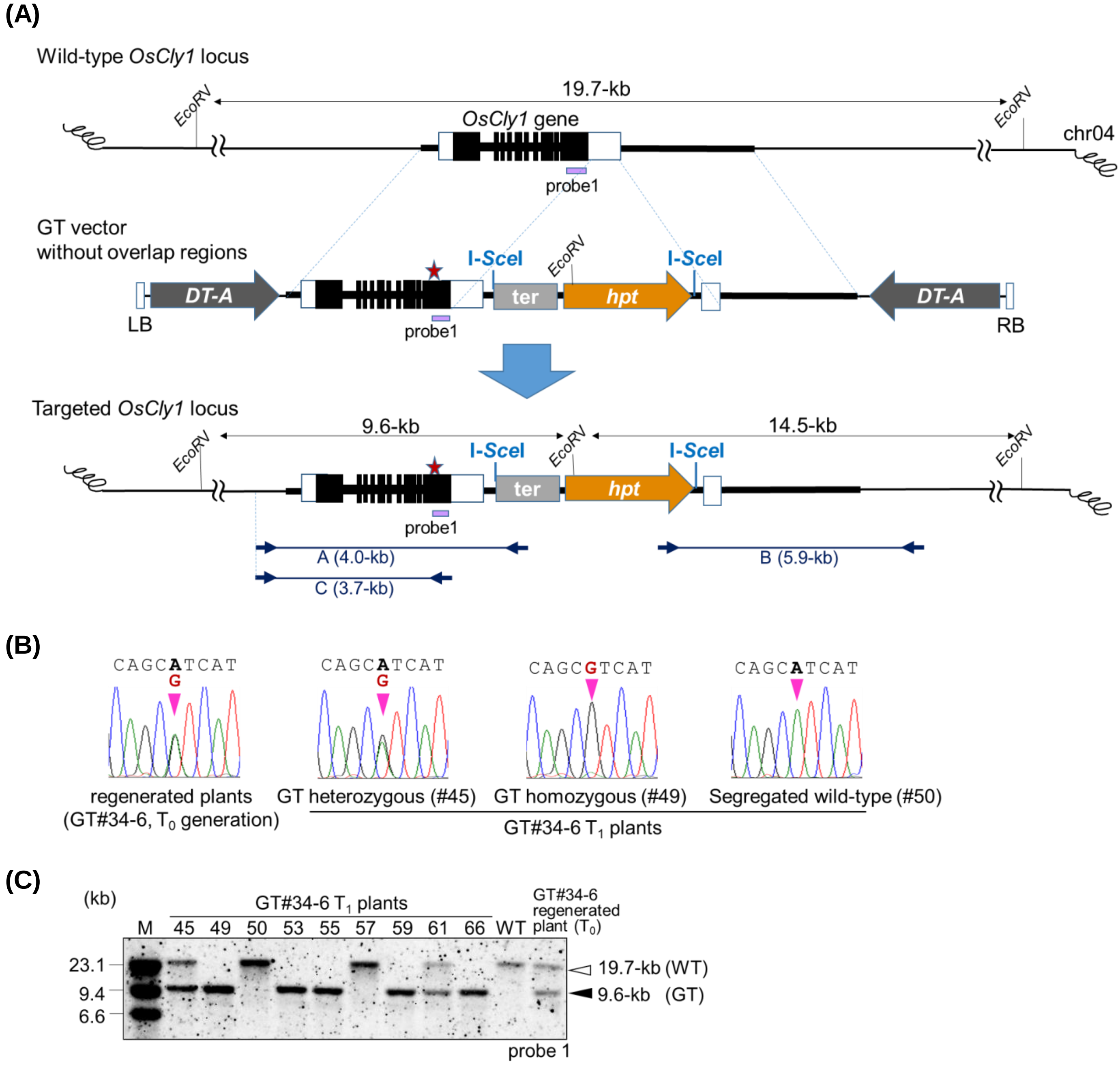

Supplementary Figure S2. GT strategy using a GT vector without overlapped region. (A) Schematic representation of GT experiments. GT scheme as in Figure 1A. A total of 179 lines of hygromycin-resistant calli were obtained from 2,069 pieces of transformed callus. Among them, independent 5 calli lines were screened as GT candidates by PCR using primer sets A and B. Regenerated plants were obtained from GT calli, GT#34. (B) Sequencing chromatograms in GT#34. (C) Southern blot analysis in T_1 generation of GT#34. Details as in Figure 2.

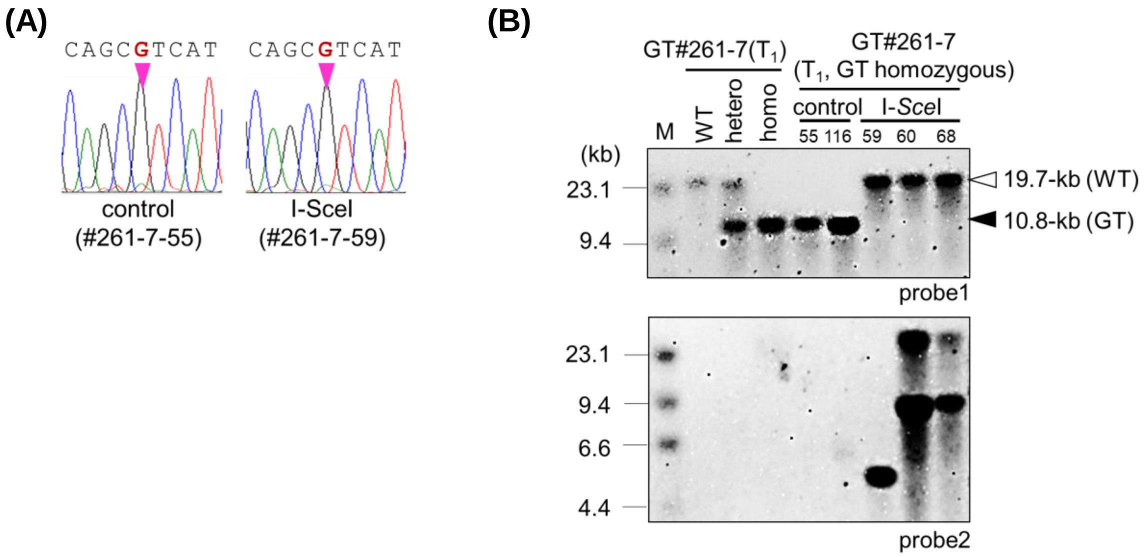

Supplementary Figure S3. Marker excision experiments via SSA in GT#261. (A) Sequencing chromatograms in regenerated plants of GT homozygous plants in T_1 generation of GT#261 transformed with control vector (left panel) or I-SceI vector (right panel). (B) Southern blot analyses using *Eco*RV- or *Msc*I-digested genomic DNA with probe1 or probe2, respectively. Samples are segregated-out wild-type (WT), GT heterozygous (hetero) and GT homozygous (homo) in T_1 plants of #GT261-7, and regenerated plants of GT homozygous in T_1 generation of #GT261-7 transformed with the control or I-SceI vector. M: size marker. Details as in Figure 3B.

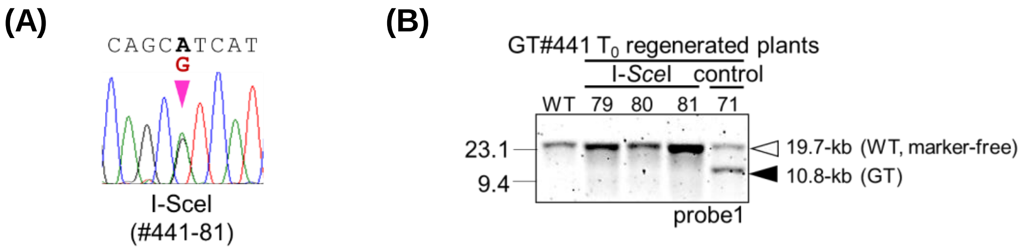

Supplementary Figure S4. GT and marker excision experiments in GT#441. (A) Sequencing chromatograms in regenerated plants of GT homozygous plants in T_1 generation of GT#441-81 transformed with I-SceI vector. Sequences were determined using fragments amplified with primer set E in Figure 1B. (B) Southern blot analyses using *Eco*RV-digested genomic DNA with probe1, respectively. Samples are regenerated plants of GT heterozygous in T_0 generation of #GT439 transformed with the control or I-SceI vector. NT; non-transformant. Details as in Figure 3B.

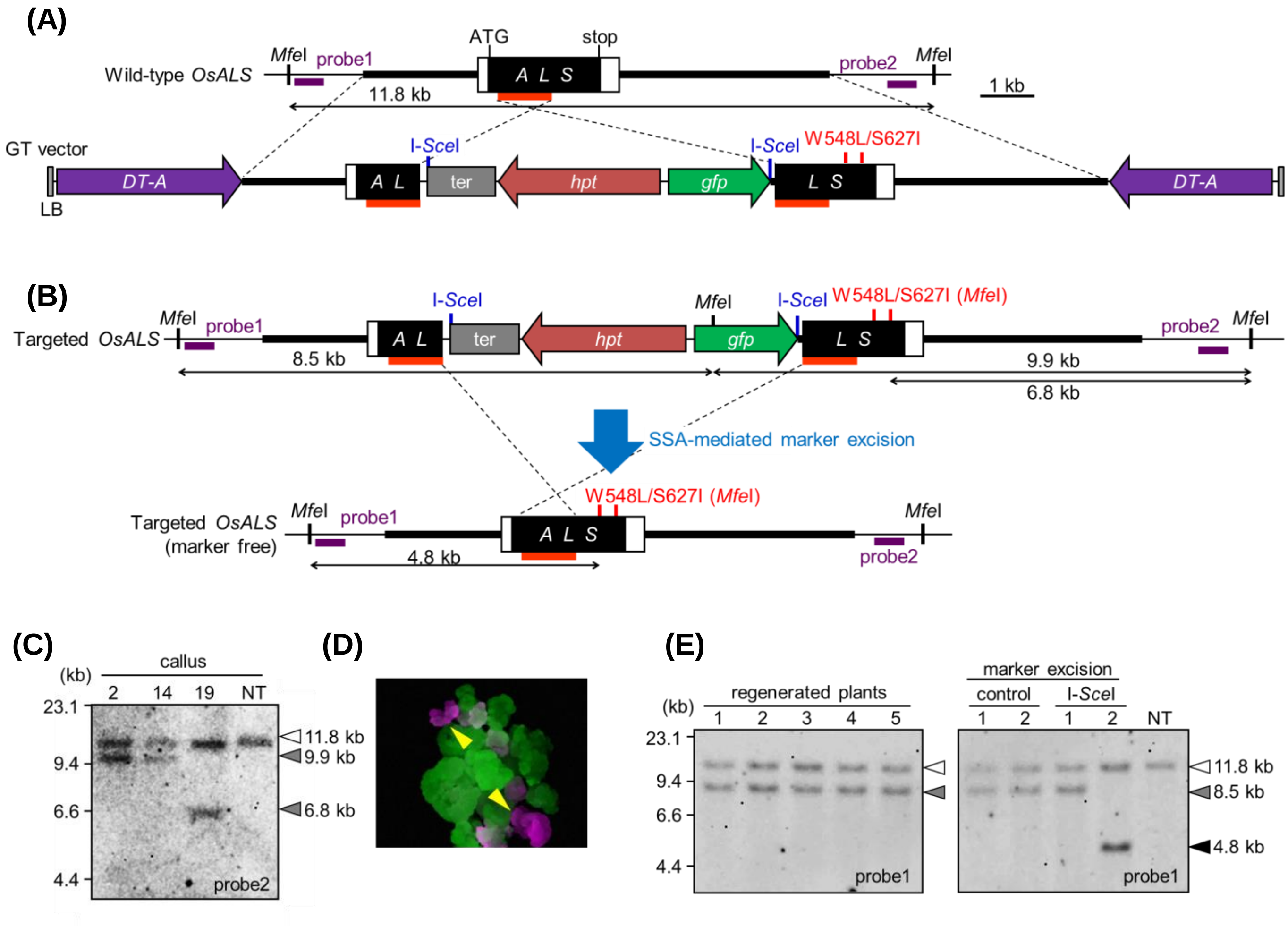

Supplementary Figure S5. Precise genome editing in *OsALS* locus.
(A and B) Schematic representation of GT (A) and marker excision (B) in *OsALS* gene. The GT vector for *OsALS* gene carries the 3.4 and 6.2-kb homology regions with point mutations [W548L; tryptophan (TGG) to leucine (TTG) at amino acid 548, S627I; serine (AGT) to isoleucine (ATT) at amino acid 627] to confer herbicide tolerance and 1.0 kb of overlapped sequences in the homology regions indicated as an orange bar. I-*Sce*I meganuclease recognition sites were located at both ends of homology sequences. Purple bars indicated probes for Southern blot analysis. hpt; *hpt* gene under the control of the double CaMV 35 promoter, gfp; *gfp* gene under the control of the 35 promoter; DT-A; *DT-A* gene under the control of the maize ubiquitin promoter or rice elongation factor promoter, ter; rice actin1 terminator, LB and RB; left and right border. (C) Southern blot analysis with probe2 shown as shown in (A) using *Mfe*I-digested genomic DNA of non-tranformant (NT) and GT calli #2, 14, and 19. (D) Image of fluorescence emitting from GT calli transformed with I-SceI vector. Green and magenta colors indicate fluorescence derived from GFP and DsRed, respectively. The image is merged with GFP and DsRed fluorescences. Yellow arrowheads show cells from which DsRed fluorescence was emitted but not GFP. (E) Southern blot analysis with probe1 using *Mfe*I-digested genomic DNA of a non-transformant, plants regenerated from GT calli #19, and plants regenerated from GT calli #19 transformed with the control vector or I-SceI vector.

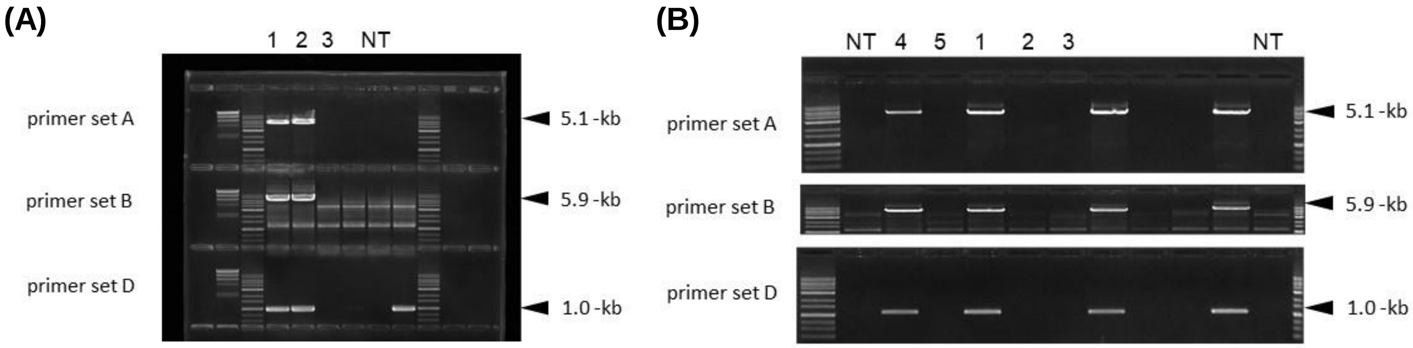

Supplementary Figure S6. Entire images of original gels.
Gel images shown in A and B are original picture of Fig. 3D and 5B, respectively.

Supplementary Table S1. Primers used in this study.

Sequence (5' – 3')	Name/Purpose
GAGAAGACGCGAGAAGACAAGCAATCGAA	To amplify 5' region of *OsCly1* in GT vector
ATGGCACCCTCGCGAGCTGGCCCCTA	
GGTTCCATTCCCTGACCCGGCCCACCT	To amplify 3' region of *OsCly1* in GT vector
TTCCCGTGGGGCAACCGCTGGGGTCT	
acagcagctgcagcGtcatcacgattccct	To substitute one base (A to G) of *OsCly1* in GT-*OsCly1* variation 1
AGGGAATCGTGATGACGCTGCAGCTGCTGT	
caacgccgcggccgccgcaaccgctacagcagcCgcagcatcatcCcga	To substitute two bases (T to C, A to C) of *OsCly1* in GT-*OsCly1* variation 2
GCTCCGAGGCGCGCCTGGTTGGTTGCCGGTGAG	
caacgccgcggccgccgcaaccgctacagcagcCgcTgcGtcGtcCcgTttTcct	To substitute seven bases of *OsCly1* in GT-*OsCly1* variation 3
GCTCCGAGGCGCGCCTGGTTGGTTGCCGGTGAG	
CGGGCGCCCACAGACCCAAAGCTAGGAC	Primer set A used in *OsCly1* GT experiments
AGAACCACAGGTAGCAATAGGTATTACAGT	
GTGCCTCCACAGGAAAACCAGCGGCAGT	Primer set B used in *OsCly1* GT experiments
TTCCCGTGGGGCAACCGCTGGGGTCT	
CGGGCGCCCACAGACCCAAAGCTAGGAC	Primer set C used in *OsCly1* GT experiments
GTCATCGTTGTTGGCCATGGTGAAAGAAA	
GCGATAAAGGAAAGGCCATCGTTGAAGA	Primer set D used in *OsCly1* GT experiments
CGTCTGCTGCTCCATACAA	
CGGGCGCCCACAGACCCAAAGCTAGGAC	Primer set E used in *OsCly1* GT experiments
ATGGCACCCTCGCGAGCTGGCCCCTA	
GGTTCCATTCCCTGACCCGGCCCACCT	To amplify Probe 1 used in *OsCly1* GT experiments
CAGTGAATGATGCAACATGAGACCGAACAA	
CCAGGTTATGAACCTCGGTCCCAACTCTAAGCTC	To amplify Probe 2 used in *OsCly1* GT experiments
TCACTTGAGGAAGGTCTCCGACGAAATGGTGTT	
TCCATGGAAATATGGGCCAAAAGCTAACAA	To confirm true GT events in 5′ region of *OsALS* locus.
CAGGAGGACGGCGATAACAGCTCCTCTTG	
CTTAAGATTGAATCCTGTTGCCGGTCTTGC	To confirm true GT events in 3′ region of *OsALS* locus.
TCGATCGTTGGATGTGTCACACGATGAAT	
TTCTTTTTCAATACTTTCCTCGCTTGCTCT	To amplify Probe 1 used in *OsALS* GT experiments
ATTCAGCCACTTATCTTGACACAACCATTT	
ACATTCATGACCCGTGAGGGCAATATGAGATA	To amplify Probe 2 used in *OsALS* GT experiments
AGTGGTCTCCCAGCAGACAGGCTGC	

Supplementary Table S2. Summary of GT experiments for *OsALS* locus.

	Fresh weight of *Agrobacterium*-infected calli	No. of Hygromycin tolerant calli	No. of GT calli by PCR analysis	No. of GT calli with desired mutations
Exp.1	12.6 g	500	3	1 (#19)
Exp.2	14.8 g	327	2	2
Total	27.4 g	827	5	3
